# Human METTL20 Methylates Lysine Residues Adjacent to the Recognition Loop of the Electron Transfer Flavoprotein in Mitochondria*

**DOI:** 10.1074/jbc.M114.580464

**Published:** 2014-07-14

**Authors:** Virginie F. Rhein, Joe Carroll, Jiuya He, Shujing Ding, Ian M. Fearnley, John E. Walker

**Affiliations:** From The Medical Research Council Mitochondrial Biology Unit, Hills Road, Cambridge CB2 0XY, United Kingdom

**Keywords:** Bioenergetics, Electron Transport System (ETS), Flavoprotein, Mitochondrial Metabolism, Protein Methylation, Electron Transfer Flavoprotein, Methyltransferase, Trimethyllysine

## Abstract

In mammalian mitochondria, protein methylation is a relatively uncommon post-transcriptional modification, and the extent of the mitochondrial protein methylome, the modifying methyltransferases, and their substrates have been little studied. As shown here, the β-subunit of the electron transfer flavoprotein (ETF) is one such methylated protein. The ETF is a heterodimer of α- and β-subunits. Lysine residues 199 and 202 of mature ETFβ are almost completely trimethylated in bovine heart mitochondria, whereas ETFα is not methylated. The enzyme responsible for the modifications was identified as methyltransferase-like protein 20 (METTL20). In human 143B cells, the methylation of ETFβ is less extensive and is diminished further by suppression of METTL20. Tagged METTL20 expressed in HEK293T cells specifically associates with the ETF and promotes the trimethylation of ETFβ lysine residues 199 and 202. ETF serves as a mobile electron carrier linking dehydrogenases involved in fatty acid oxidation and one-carbon metabolism to the membrane-associated ubiquinone pool. The methylated residues in ETFβ are immediately adjacent to a protein loop that recognizes and binds to the dehydrogenases. Suppression of trimethylation of ETFβ in mouse C2C12 cells oxidizing palmitate as an energy source reduced the consumption of oxygen by the cells. These experiments suggest that the oxidation of fatty acids in mitochondria and the passage of electrons via the ETF may be controlled by modulating the protein-protein interactions between the reduced dehydrogenases and the β-subunit of the ETF by trimethylation of lysine residues. METTL20 is the first lysine methyltransferase to be found to be associated with mitochondria.

## Teaser

**Background:** The electron transfer flavoprotein (ETF) is a mobile electron carrier coupling mitochondrial fatty acid oxidation to respiration.

**Results:** METTL20 is a mitochondrial lysine protein methylase that modifies Lys-199 and -202 of the electron transfer flavoprotein β-subunit.

**Conclusion:** METTL20 is a protein methylase in mitochondria that influences protein-protein interactions with ETF.

**Significance:** Mitochondria contain lysine methyltransferase METTL20.

## Introduction

The post-translational methylation of proteins by methyltransferases with *S*-adenosylmethionine as the methyl donor occurs primarily on the side chains of lysine and arginine residues, but histidyl and glutamyl side chains and α-amino and α-carboxyl groups can be methylated also (1, 2). The ϵ-amino groups of lysine residues can carry one, two, or three methyl groups, and the guanidino moieties of arginines can be monomethylated, and dimethylated either symmetrically or asymmetrically. The remodeling of chromatin by methylation and demethylation of lysine and arginine residues in histones, together with acetylation and deacetylation of lysines, influences gene expression, DNA replication, and DNA repair and apoptosis among other processes (3–7), and methylation of lysine and arginine residues in non-histone proteins is being associated increasingly with the regulation of many other cellular activities (8, 9). Moreover, lysine residues can also be ubiquitinated and sumoylated, and the interplay between methylation, acetylation, and these other post-translational modifications, at the same and neighboring sites, adds complexity to the regulation of biological processes by post-translational modification (9–11). The methylation reactions are catalyzed by methyltransferases, and 30% of known and putative methyltransferases are associated with diseases including cancer, inflammation, and metabolic disorders (12–14).

Hitherto, the methylation of lysine and arginine residues of proteins found in mitochondria has been little investigated. It is well known that lysine residues in apocytochrome *c* are methylated by Ctm1p in the cytoplasm of *Saccharomyces cerevisiae* (15, 16), but this modification has no clear role, and the mammalian orthologue is unmodified. Trimethyllysine residues have been characterized in three proteins isolated directly from mammalian mitochondria: they are citrate synthase (17), ADP/ATP translocase (18), and the c-subunit in the rotor of ATP synthase (19). A dimethylarginine residue has been characterized in the NDUFS2 subunit of complex I (20), and a complex pattern of methylation of three histidine residues has been found near the N terminus of the NDUFB3 subunit of complex I (21). In addition to these five methylations characterized in proteins purified from mitochondria, other human proteins that locate to mitochondria have been reported in cell-wide methylome studies to contain methylated lysine and arginine residues (22, 23).

With the exceptions of HEMK1^3^ (heme biosynthesis gene K) and NDUFAF7, the mammalian methyltransferases responsible for the modifications of mitochondrial proteins, their subcellular locations, and the biological significance of the modifications are unknown. HEMK1 methylates a glutamine residue in the mitochondrial translation release factor MTRF1L (24), and NDUFAF7 symmetrically dimethylates Arg-85 in the mature NDUFS2 subunit of the multisubunit enzyme, complex I, an essential step in the assembly of the complex (25). NDUFAF7 is one of 35 known and possible mitochondrial methylases catalogued previously by bioinformatic analysis (25).

As described below, we have found that another mitochondrial protein, the β-subunit of the electron transfer flavoprotein (ETF) is also methylated. The ETF is a heterodimeric complex of α- and β-subunits, known as ETFα and ETFβ, respectively (26–29). It acts as a mobile electron carrier in the matrix of mitochondria, linking 11 different mitochondrial FAD-containing acyl-CoA dehydrogenases involved in fatty acid β-oxidation to the ubiquinone pool of the respiratory chain (30, 31). The ETF also accepts electrons from sarcosine dehydrogenase and dimethylglycine dehydrogenase, two enzymes of mitochondrial one-carbon metabolism (32). A “recognition loop” in ETFβ recognizes and interacts with these 13 different dehydrogenases (33, 34), and the sites of methylation are immediately adjacent to the recognition loop. Once bound to the recognition loop, the dehydrogenases reduce an FAD prosthetic group in the ETF. Then the reduced ETF carries the electrons to the ETF-quinone oxidoreductase bound in the inner mitochondrial membrane (35). The precise mode of interaction of ETF with the quinone oxidoreductase is not known, but ETFβ is involved in the formation of this complex (36). Finally, the reduced ETF-quinone oxidoreductase transfers the electrons to ubiquinone, and the reduced quinone enters the quinone pool, providing electrons for the cytochrome *bc*_1_ complex (complex III) to reduce cytochrome *c*.

The location of the methylated lysine residues characterized in the current study, immediately adjacent to the recognition loop, suggests that their methylation may influence the interactions of the ETF with the dehydrogenases and possibly with ETF-quinone oxidoreductase, and that it could provide a mechanism of regulating the β-oxidation of fatty acids. We have also identified METTL20 (methyltransferase-like protein 20) as the mitochondrial methyltransferase that introduces methyl groups into two specific lysine residues in ETFβ. No protein lysine methyltransferase had been characterized previously in mitochondria.

## EXPERIMENTAL PROCEDURES

### 

#### 

##### Cell Culture

Human 143B osteosarcoma cells (ATCC number CRL8303) and mouse C2C12 myoblasts (Public Health England 91031101) were grown at 37 °C in Dulbecco's modified Eagle's medium (DMEM) containing 25 mm glucose and supplemented with fetal bovine serum (FBS, 10% v/v), penicillin (100 units/ml), and streptomycin (0.1 mg/ml) under an atmosphere of 5% CO_2_. The serum in the media for parental human embryonic kidney cells (HEK293T) and FLAG-strepII-tagged METTL20 inducible HEK293T cells was tetracycline-free, and the media included blasticidin (15 μg/ml) and zeocin (100 μg/ml), or blasticidin (15 μg/ml) and hygromycin (100 μg/ml), respectively.

##### Plasmid Transfection and Confocal Microscopy

The cDNA for human METTL20 (Thermo Scientific, Loughborough, UK) was amplified by PCR with the forward and reverse primers: 5′-CGCGGATCCGGAATGGCTTTGAGTCTAGGTTGGAAAG-3′ and 5′-ATAGTTTAGCGGCCGCCCAGGCTGAAAACCCCACACTGTGC-3′, respectively. It was cloned into the inducible expression vector pcDNA5/FRT/TO (Invitrogen) with sequences encoding C-terminal FLAG and Strep tags, and incorporated stably into HEK293T Flp-In^TM^ T-Rex^TM^ cells. Plasmids were transfected into 143B cells with Lipofectamine 2000 (Invitrogen). After 24 h, the mitochondria were labeled with MitoTracker Orange (200 nm; Invitrogen), and the nuclei were stained with DAPI (0.4 μg/ml). The same cells were fixed with paraformaldehyde (4%, w/v) and permeabilized with Triton X-100 (0.5%, v/v). The FLAG-tagged METTL20 was detected with mouse M2 anti-FLAG antibody (Sigma) followed by Alexa Fluor 488 goat anti-mouse secondary antibody (Invitrogen). The fluorescent signal was visualized with a Zeiss 510 LSM confocal microscope (Zeiss, Cambridge, UK).

##### Preparation of Bovine ETF

Submitochondrial particles were prepared from bovine heart mitochondria (37). They were centrifuged (37,000 × *g* for 20 min, and then at 110,000 × *g* for 30 min), and the supernatant was fractionated at room temperature by ammonium sulfate precipitation. The 60–90% fraction was re-solubilized in buffer A (pH 7.4) consisting of 25 mm HEPES and 0.1 mm EDTA, dialyzed against the same buffer, and fractionated by cation exchange chromatography on a HiTrap SP HP column (1 ml; GE Healthcare Bio-Sciences AB, Uppsala, Sweden). The sample was loaded onto the column equilibrated in buffer A at a flow rate of 0.5 ml/min, and eluted with a linear gradient of 0–150 mm NaCl in buffer A over 60 min at room temperature. Fractions containing the partially purified ETF were analyzed by SDS-PAGE and mass spectrometry.

##### Expression and Affinity Purification of Tagged METTL20

Plasmid pcDNA^TM^5/FRT/TO encoding METTL20 with C-terminal StrepII and FLAG tags was cotransfected in the presence of Lipofectamine 2000 (Invitrogen) with plasmid pOG44 into human HEK293T Flp-In T-Rex cells (total DNA, 1 μg; pOG44:pcDNA5/FRT/TO, 7:1 by weight) (Invitrogen) (38). After 24 h, the medium was replaced with the selective medium containing blasticidin (15 μg/ml) and hygromycin (100 μg/ml) and inducible cell lines expressing the recombinant protein were selected. Expression of tagged METTL20 was induced for 24 h with doxycycline (20 ng/ml). Mitochondria were prepared (38) and solubilized in 1% (w/v) *n*-dodecyl β-d-maltoside for affinity purification of tagged METTL20 and associated proteins. To prepare mitoplasts, cells were suspended at a protein concentration of 10 mg/ml in PBS-inhibitor (phosphate-buffered saline with complete EDTA-free protease inhibitor from Roche Applied Science) and enriched for mitoplasts by addition of an equal volume of digitonin (1 mg/ml) in PBS-inhibitor to give a detergent:protein ratio of 1:10 (w/w) (39). The sample was centrifuged (11,000 × *g*, 5 min, 4 °C), and mitoplasts were solubilized from the pellet with 1% (w/v) *n*-dodecyl β-d-maltoside. The enriched mitochondrial protein preparation was loaded onto a StrepII tag gravity column (Pierce Spin Column containing Strep-Tactin-Sepharose) followed by 5 column volumes of wash buffer (20 mm HEPES, pH 7.6, 0.2 mm EDTA, 150 mm NaCl, 2 mm dithiothreitol, 0.1 mm phenylmethylsulfonyl fluoride, Roche protease inhibitor (1/50; v/v), and 0.05% (w/v) *n*-dodecyl β-d-maltoside). Bound protein was eluted with 6 portions of 0.5 column volumes of wash buffer containing 10 mm desthiobiotin. Eluates 1–3 were combined and analyzed by SDS-PAGE and mass spectrometry.

##### Suppression of Expression with siRNA

Transcripts of METTL20 were suppressed transiently in human 143B cells with 30 nm siRNAs and transcripts of METTL20 or ETFβ in mouse C2C12 cells with 100 nm siRNAs (Sigma). Allstars negative control siRNA (Qiagen, Crawley, UK) was used at the same concentration as the specific siRNA. Two transfections were performed at 0 and 72 h. The levels of the transcripts (normalized to endogenous β-actin) were investigated at 48 and 120 h by quantitative real-time PCR performed with TaqMan gene expression assays (Invitrogen) on cDNA prepared with a Cells-to-CT kit (Invitrogen). Mitoplasts were prepared 48 h after each transfection, and the oxygen consumption rate (OCR) of C2C12 cells was assessed 48 h after the second transfection.

##### Protein Analyses

Protein concentrations were estimated by bicinchoninic acid assay (Pierce, ThermoFisher). Proteins were fractionated by SDS-PAGE on Novex Tris glycine 10–20% acrylamide gradient gels (Invitrogen) and transferred electrophoretically to an Immobilon P membrane (Millipore, Billerica, MA) for immunodetection. The membrane was washed in PBS containing 0.01% (v/v) Tween 20 (PBST), and then treated with a solution of dried skimmed milk, or where anti-methyl antibodies were to be employed, with bovine serum albumin (3%, w/v) in the same buffer. Bound proteins were reacted with primary rabbit antibodies with specificities for dimethyllysine and trimethyllysine (Abcam, Cambridge, UK) for ETFβ and citrate synthase (Proteintech, Manchester, UK) and NDUFB8 (Sigma). Bound rabbit antibodies were peroxidase conjugated with goat anti-rabbit antiserum (ThermoFisher), and detected with enhanced chemiluminescence reagents (GE Healthcare).

##### Mass Spectrometry

Bovine, human, and mouse proteins in extracts and column fractions were reduced, alkylated, and fractionated by SDS-PAGE in 12–22 or 10–20% polyacrylamide gradient gels, as described before (25). Proteins were characterized by tandem-MS analysis of tryptic, chymotryptic, and AspN digests of Coomassie Blue-stained bands or sections of gel. Extracted peptides were analyzed in a MALDI-TOF-TOF mass spectrometer (model 4800; AB-Sciex, Warrington UK) with α-cyano-4-hydroxycinnamic acid as matrix, or in an LTQ OrbiTrap XL-ETD (electron transfer dissociation) mass spectrometer (Thermo Scientific) coupled to a Proxeon Easy-nLC (Thermo Scientific) with a C_18_ column (100 mm × 75 μm inner diameter; Nanoseparations, Nieukoop, The Netherlands) for reverse-phase fractionation of peptides using an acetonitrile gradient in 0.1% (v/v) formic acid with a solvent flow rate of 300 nl/min. Peptides were fragmented by collision-induced dissociation with either air (4800 instrument) or nitrogen (LTQ OrbiTrap XL-ETD), or by ETD with fluoranthene radical anions (LTQ OrbiTrap XL-ETD) and supplemental activation (40). MALDI-TOF-TOF data were analyzed with Mascot (41) (version 2.4.0) using the following parameters: NCBInr database 20120611, taxonomy, Mammalia; precursor ion mass tolerance 70 ppm; fragment ion mass tolerance 0.8 Da; Met oxidation variable, Cys-carbamidomethyl fixed (iodoacetamide-treated samples) or Cys-propionamide (non-alkylated samples); trypsin 2-missed cleavages. A significance threshold of *p* < 0.05 was used for peptide identification. Orbitrap peptide fragmentation data were analyzed using Proteome Discoverer 1.3 (Thermo Fisher) with Mascot and Peptide Validator nodes. The following parameters were employed: UniProt_2013_04, taxonomy, Mammalia (for bovine and mouse)/*Homo sapiens* (for human); precursor ion mass tolerance 5 ppm; fragment ion mass tolerance 0.5 Da; variable modifications, Met oxidation, Lys/Arg-methyl, Lys/Arg-dimethyl, Lys-trimethyl; fixed modification Cys-carbamidomethyl; trypsin 2-missed, AspN_ambic 3-missed, chymotrypsin 4-missed cleavages; decoy database search (false discovery rate values 0.01 and 0.05). The fragmentation spectra of modified peptides were interpreted manually. ETD fragment ions were assigned as *c* and *z* ions (including related *z*+H ions) (42). The relative abundance of methylation of ETFβ was determined by analysis of an AspN-generated peptide (EPRYATLPNIMKAKKKKI) that contains both methylated lysine residues. Monoisotopic *m*/*z* values used are 710.4263 and 715.7579 (M + 3H)^3+^, plus 533.0717 and 537.0704 (M + 4H)^4+^ for the non-methylated and non-methylated/Met-oxidized forms of the peptide, respectively. 14.0156 Da were added for each methyl group. The peak areas of Gaussian smoothed extracted ion chromatograms, with a *m*/*z* tolerance of 5 ppm and overlapping peptide retention times, were obtained with the aid of Xcalibur software. The intact bovine ETF subunits were separated by reverse-phase chromatography and their molecular masses were determined by LC-MS (43).

##### Protein Quantitation of SILAC-labeled Samples

Inducible HEK293T cells expressing either tagged METTL20 or METTL12 were grown in “heavy” DMEM containing arginine and lysine isotopically labeled with ^15^N and ^13^C, and in “light” DMEM containing ^14^N- and ^12^C-arginine and -lysine (Sigma) (44). These media were supplemented with penicillin (100 units/ml) and streptomycin (0.1 mg/ml), and proline (200 mg/liter) to suppress the conversion of arginine to proline, and with dialyzed FBS (10% v/v) (Invitrogen) to prevent dilution of heavy isotopes. To ensure maximal incorporation, the cell population was doubled at least seven times.

Cells were mixed 1:1 (protein w/w) and consisted of at least 10 mg of protein from each of the heavy and light isotope-labeled cultures. Then mitoplasts were prepared and samples for analysis consisted of StrepII-tag affinity-purified proteins as described under “Expression and Affinity Purification of Tagged METTL20.” The protein samples were fractionated by SDS-PAGE, and Coomassie Blue-stained gel slices were subject to in-gel trypsin digestion and analysis in an LTQ Orbitrap XL-ETD as described above. Each mass spectrometric ratio was based on two SILAC analyses. In the first orientation, the cell line over-expressing METTL20 was labeled in heavy medium and a control cell line in which METTL12 was overexpressed in light medium, and vice versa in the second orientation. Protein identification and relative quantification was performed with MaxQuant and the integrated Andromeda search engine, with the Perseus program used for subsequent bioinformatic and statistical analyses (45–47). Heavy and light peptide pairs were quantified with MaxQuant (version 1.3.0.5) and the following parameters used: Fasta database: IPI Human (version 3.68), oxidation (M) and acetyl (protein N-term) variable; Cys-carbamidomethyl fixed; MS/MS balance 0.5 Da; minimum ratio count 2; Arg-10 and Lys-8. The median peptide ratio was taken to be the protein ratio, using at least two values. The ratios from each experiment were plotted on horizontal and vertical axes, respectively, of a “scatter plot” as the log base 2 value. Thus, a 4-fold change in the abundance of a protein in both experiments becomes 2 on each axis and the protein is represented by a point in the top right or bottom left quadrant of the scatter plot. Proteins unaffected by the experimental condition are clustered around the intersection of the *x* and *y* axes. A diagonal line extending from the top right to bottom left quadrants represent a perfect correlation between the two experiments. Statistically significant proteins in both orientations of labeling were identified with Perseus (*p* < 0.05). Points in the two other quadrants represent proteins where the quantification differences are not reproducible in the replicate experiments. Those in the top left quadrant contain unlabeled external contaminants such as keratins or dermcidin. SILAC experiments were performed twice with each labeling orientation. A comprehensive account of the SILAC methodology and data analysis used here has been described previously (45–47).

##### Measurement of Respiration

Mouse C2C12 cells were transfected twice at 0 and 72 h, with 100 nm siRNA of negative control, METTL20, or ETFβ. The OCR of transfected cells was measured in an XF96 extracellular flux analyzer (Seahorse Biosciences, MA) at 120 h. The assay medium consisted of 70 mm sucrose, 220 mm mannitol, 10 mm KH_2_PO_4_, 5 mm MgCl_2_, 2 mm HEPES, 1 mm EGTA, 0.2% (w/v) fatty acid-free BSA (pH 7.4), and 4 mm ADP. The cells were treated with 1 nm XF plasma membrane permeabilizer reagent (Seahorse Biosciences). When the OCR reached a stable baseline, 40 μm palmitoyl-l-carnitine chloride supplemented with 0.2 mm malate was added. Respiration was normalized to cell number by the sulforhodamine B colorimetric assay (48). *p* values for the OCR were calculated with a paired Student's *t* test.

##### Structural Analyses

The mitochondrial targeting sequence of METTL20 was predicted with Mitoprot (49) and its secondary structure with Jpred3 (50). Sequences were aligned with ClustalW. The structure of the region of interaction between the recognition loop in recombinant human ETFβ and MCAD (33) was produced with PyMol using Protein Data Bank code 1t9g (51).

## RESULTS

### 

#### 

##### Mitochondrial Methyltransferases

**FIGURE 1.**
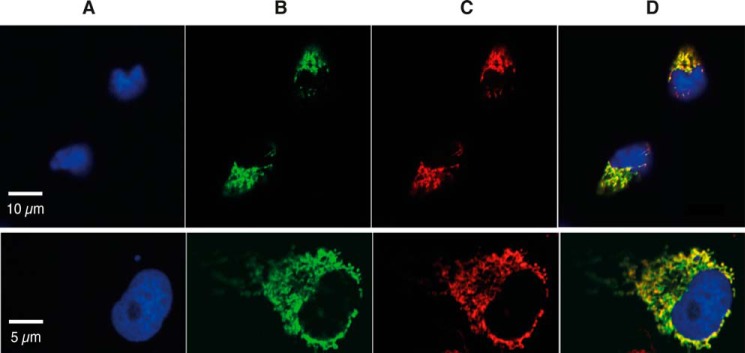
**Subcellular location of METTL20 and METTL12.** METTL20 or METTL12, each with C-terminal StrepII and FLAG tags were expressed in human 143B cells (*upper* and *lower panels*, respectively). *A*, cell nucleus stained with DAPI (*blue*). *B*, recombinant METTL20 or recombinant METTL12 detected with an anti-FLAG antibody, plus goat anti-mouse Alexa Fluor 488 (*green*). *C*, mitochondria stained with MitoTracker (*red*). *D*, merged areas of *A–C*.

Previously, a catalogue of 35 known and possible mitochondrial methyltransferases was compiled (25). In this list, the functions of seven established mitochondrial proteins have been demonstrated: four are mitochondrial RNA methyltransferases, NDUFAF7 is a mitochondrial arginine-methyltransferase, and two others are involved in ubiquinone biosynthesis. To this catalogue can be added the catechol *O-*methyltransferase and catechol *O-*methyltransferase domain containing protein-1 (52, 53), and the N5 glutamine methyltransferase, HEMK1 (24). The list is dominated by the family of 7β-strand methyltransferases (25). As part of a wider investigation of protein methyltransferases in mitochondria, one highly conserved member of the 7β-strand family, METTL20, was chosen for study. Another possible methyltransferase, METTL12 (methyltransferase-like protein 12), was used as a control in experiments employing SILAC designed to identify binding partners of METTL20 (see below). Both METTL12 and METTL20 localized to the mitochondria of human 143B cells (Fig. 1).

##### Interaction between METTL20 and ETF

**FIGURE 2.**
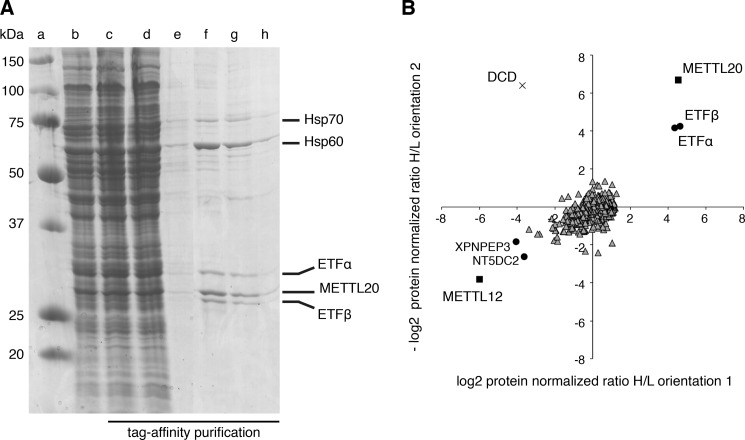
**Identification of proteins associated with METTL20.** *A,* SDS-PAGE analysis of METTL20 and associated proteins purified by affinity chromatography from Flp-In T-REx HEK293T cells expressing FLAG-StrepII-tagged METTL20. *Lane a*, molecular mass marker proteins (molecular masses indicated on the *left*); *lane b*, solubilized mitochondria; *lane c*, unbound proteins; *lanes d* and *e*, first and final column washes; *lanes f–h*, eluates 1, 3, and 5; on the *right* of the gel are shown the identities of the stained bands determined by tandem-MS analysis of tryptic peptides from the bands in *lanes f* and *g. B*, quantitative mass spectrometry of METTL20 and associated proteins. SILAC-labeled cells from METTL20 and differentially labeled control cells overexpressing METTL12 were combined and purified as above. The experiment was performed in both labeling orientations (orientation 1, METTL20-heavy plus METTL12-light; orientation 2, METTL20-light plus METTL12-heavy). Each data point corresponds to the abundance ratio of an identified protein from the two complementary experiments. ■, METTL20 and METTL12; ●, proteins significantly associated with METTL20 or METTL12 in both orientations; ▵, 721 proteins that comprise 709 insignificantly associated with either tagged-protein (including HSP70) and 12 proteins with one significant orientation only, including HSP60; ×, exogenous contaminant (DCD, dermcidin).

Four proteins, ETFα, ETFβ, and the heat shock proteins, HSP60 and HSP70, were found associated with a tagged version of METTL20 (Fig. 2*A*). Authentic binding partners were distinguished from nonspecifically interacting proteins and exogenous contaminants, by quantitative mass spectrometry of SILAC-labeled samples (Fig. 2*B*). This experiment confirmed that ETFα and ETFβ were associated significantly with METTL20. There was also a suggestion of a possible association between METTL20 and HSP60, but the experiment provided no support for the possibility of a specific interaction between METTL20 and HSP70. In other experiments (not shown), no evidence was found for the methylation of human HSP60.

##### Methylation of the β-Subunit of the ETF in Bovine and Human Mitochondria

**FIGURE 3.**
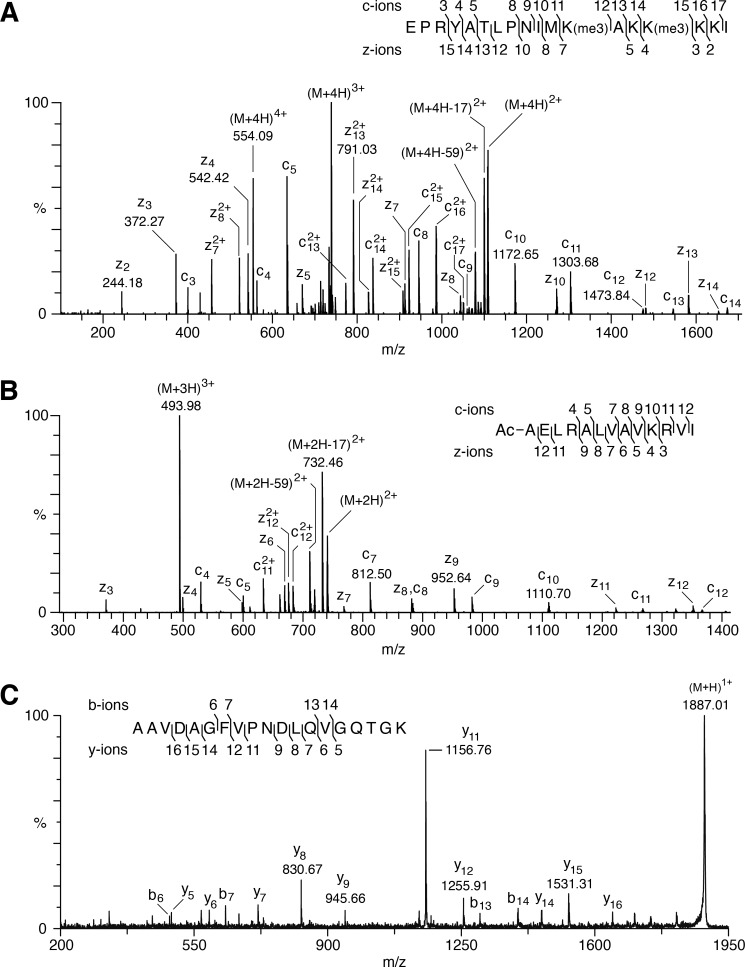
**Post-translational modifications of bovine ETFβ and the presence of proline at position 239 of bovine ETFα.** *A*, ETD fragmentation mass spectrum of peptide DN-(188–205) of ETFβ from bovine heart mitochondria, with series of *c* ions and *z* ions generated from the [M + 4H]^4+^ precursor ion, *m*/*z* 554.09, mapped onto the sequence of the peptide. *B,* sequence of the N-terminal peptide of bovine ETFβ. An ETD fragmentation mass spectrum is shown of a triply charged ion, *m*/*z* 493.98, generated by cleavage of the protein with AspN. In the *inset*, the series of *c* and *z* fragment ions identified the peptide as the N-terminal fragment, and demonstrated that the α-amino group of Ala-1 is acetylated. *C,* a MALDI-TOF-TOF fragmentation mass spectrum is shown of a singly charged ion, *m*/*z* 1886.96, of a tryptic peptide from the bovine ETF α-subunit. In the *inset*, the series of *b* and *y* fragment ions shows that the peptide corresponds to residues 231–249, and the *y*_9_ and *y*_11_ ions define the sequence Pro-Asn, and demonstrates the presence of proline and not threonine at position 239.

**FIGURE 4.**
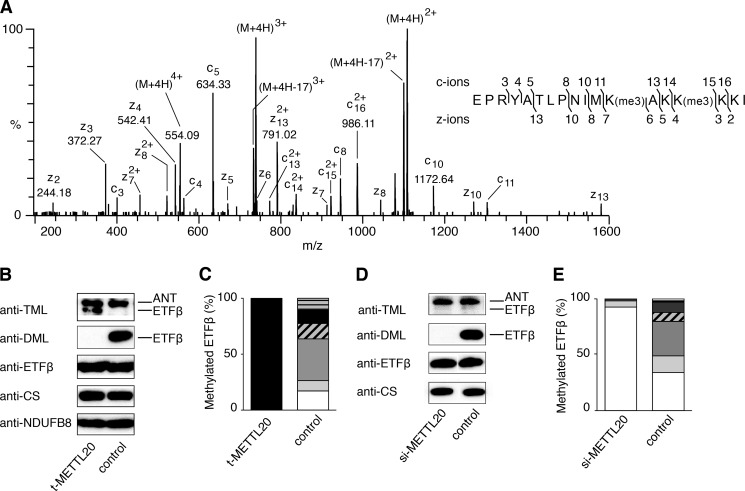
**Trimethylation of lysines 199 and 202 of human ETFβ and influence of METTL20 on their methylation.** *A–C*, samples of mitoplasts from human HEK293T cells expressing tagged METTL20 (*t-METTL20*), and from control cells. *A*, ETD fragmentation mass spectrum of peptide DN-(188–205) of ETFβ from t-METTL20 cells, with a series of *c* ions and *z* ions generated from the [M + 4H]^4+^ precursor ion mapped onto the sequence of the peptide. *B*, fractionation of mitoplasts by SDS-PAGE and Western blotting with antibodies against TML, DML, ETFβ, citrate synthase (*CS*), and the NDUFB8 subunit of complex I, employed as loading controls. *C*, quantitation of methylation states of the DN-(188–205) peptide. The methylation states of the individual Lys-199 and Lys-202 were not resolved. The histograms were derived from the extracted ion chromatograms for the *m*/*z* values of all possible methylation states of DN-(188–205). The *white*, *pale gray*, *gray*, *diagonal striped*, *dark gray*, *horizontal striped,* and *black* sections represent 0–6 methyl groups, respectively, in the peptide. *D* and *E*, human 143B osteosarcoma cells were transfected twice at 0 and 72 h with siRNA specific for METTL20 (*si-METTL20*) and negative control siRNA, and mitoplasts were prepared from the cells at 120 h. *D,* fractionation of mitoplasts by SDS-PAGE, and Western blotting with antibodies against TML, DML, ETFβ, and CS employed as a loading control. *E,* quantitation of methylation states of DN-(188–205). For an explanation of the shading, see the legend to *panel C.* In the Western blots, control 143B and HEK293T cells exhibit a stronger and more specific signal with a DML antibody than with an antibody against TML reflecting the less extensive methylation of ETFβ in these cells.

The methylation status of the α- and β-subunits of the readily available bovine ETF was examined by measuring their molecular masses (Table 1), and by verifying the sequences by analysis of proteolytic peptides. The mass of an AspN peptide (referred to as DN-(188–205)) containing five lysine residues and representing residues 188–205 of mature ETFβ indicated that the protein was methylated in this region, and a spectrum arising from the fragmentation by ETD of a quadruply charged ion of this peptide with *m*/*z* 554.1 revealed that lysine residues 199 and 202 were trimethylated (92% at both sites), whereas lysine residues 201, 203, and 204 were unmodified (Fig. 3*A*). The accurate peptide mass (M + H 2213.3571) showed that the modification at each site was trimethylation (M + H 2213.3571) and not acetylation (M + H 2213.2843). There was no evidence for non-methylated or singly methylated species, but 7.8% of the peptide carried five methyl groups and the remaining 0.2% was accounted for by forms with the two lysine residues jointly carrying two, three, and four methyl groups. ETFβ has no processed N-terminal mitochondrial targeting sequence (54), and analysis of the N-terminal AspN peptide showed that the translational initiator methionine residue had been removed, and that the resulting N-terminal alanine residue had been Nα-acetylated (Fig. 3*B*). When this modification and the presence of the two trimethylated lysine residues are taken into account, the intact molecular mass of ETFβ agrees closely with the calculated value (Table 1). In contrast, no methylated amino acid was detected in proteolytic digests of mature ETFα including Arg-204 reported recently to be monomethylated (23). The experimentally determined mass of intact ETFα was accounted for by the presence of proline at position 239 (Fig. 3*C*), and the removal of the 19-amino acid predicted N-terminal mitochondrial targeting sequence (49, 54) followed by cyclization of the resulting N-terminal Gln residue to pyroglutamate. The sequence of DN-(188–205) from human ETFβ is identical to that of the bovine peptide, and lysine residues 199 and 202 were trimethylated also (Fig. 4*A*). These residues are highly conserved (34).

**TABLE 1:** **Molecular masses of bovine mitochondrial ETFα and ETFβ** A protein fraction containing ETF was prepared from bovine mitochondrial matrix by ammonium sulfate precipitation and cation exchange chromatography. It was separated by reverse-phase chromatography on an mRP column (75 × 1 mm inner diameter, 5-μm particles) and the protein molecular masses were determined by LC-MS (43).

Subunit	Mass (Da)			PTMs	Difference*^b^*
	Observed	Calculated			
		Unmodified	Modified*^a^*		
α	32,798.2	34,957.5	32,795.9*^c^*	Δ1–19; Gln-1 cyclized to pyro-Glu	+2.3
β	27,695.4	27,699.5	27,694.5	- Initiator Met; αN-acetyl-Ala-1; Lys-199 and Lys-202 trimethylated	+0.9

*^a^* Calculated after applying the post-translational modifications (PTMs) to the unmodified sequence, Δ1–19 denotes the N-terminal mitochondrial targeting sequence for ETFα that is removed after import.

*^b^* Difference (Da) between the observed molecular mass value and the calculated modified mass.

*^c^* Residue 239 of mature bovine ETFα has been reported to be either proline or threonine; only proline was observed.

##### Effect of Overexpression and Suppression of Expression of METTL20

The overexpression of tagged METTL20 in human HEK293T cells was accompanied by a significant increase in the trimethylation of ETFβ (without effect on the methylation of the adenine nucleotide carrier), as indicated by a strong cross-reactivity with a trimethyllysine (TML)-specific antibody and an absence of cross-reactivity with a dimethyllysine antibody (DML) (Fig. 4*B*). Mass spectrometry analyses of ETFβ demonstrated that lysines 199 and 202 were both trimethylated completely in these cells (Fig. 4*C*). In control cells, the trimethylation of ETFβ was less extensive and the protein exhibited strong cross-reactivity only with the DML-specific antibody (Fig. 4*B*), and there was no evidence of complete methylation of both lysine residues; 17.2% of DN-(188–205) was unmethylated and 9.2, 37.4, 13.6, 11.8, and 10.8% of the peptide carried 1–5 methyl groups, respectively (Fig. 4*C*).

In a complementary experiment, the expression of METTL20 was suppressed transiently in human 143B cells. After 120 h, the level of the METTL20 transcript had been reduced by at least 70% (data not shown). The protein level of ETFβ was unaltered in these cells, and there was no evidence of methylation in Western blotting experiments using DML and TML antibodies, in contrast to control cells, which showed only a strong anti-DML reactivity (Fig. 4*D*). Mass spectrometric analysis of DN-(188–205) showed that suppression of expression of METTL20 was accompanied by 92.4% of ETFβ becoming unmethylated; 5.6% was monomethylated, and 2% carried 2–4 methyl groups (Fig. 4*E*).

##### Influence of the Methylation of ETFβ on the Oxidation of Fatty Acids

**FIGURE 5.**
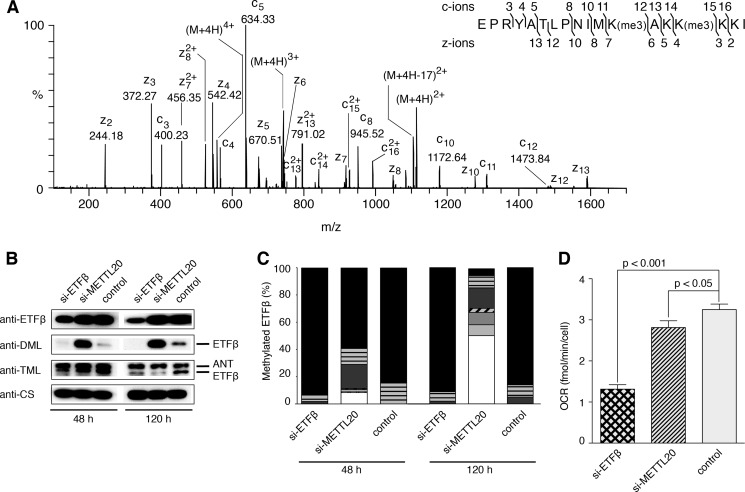
**Effect of suppression of expression of METTL20 on the methylation of ETFβ and the oxidation of palmitoylcarnitine in mouse C2C12 cells.** Mouse C2C12 myoblasts were transfected at 0 and 72 h with specific siRNAs for ETFβ and METTL20 (si-ETFβ and si-METTL20, respectively), and with negative control siRNA. *A*, ETD fragmentation mass spectrum of peptide DN-(188–205) of ETFβ from mouse C2C12 control cells, with a series of *c* ions and *z* ions generated from the [M + 4H]^4+^ precursor ion mapped onto the sequence of the peptide. *B*, mitoplasts prepared at 48 and 120 h fractionated by SDS-PAGE, and Western blotted with antibodies against ETFβ, DML, TML, and citrate synthase (*CS*). *ANT*, adenine nucleotide translocase. *C*, quantitation of methylation states of mouse DN-(188–205). For an explanation of the shading, see the legend to Fig. 4*C. D*, the cells were transfected with siRNA at 0 and 72 h. The *OCR* was measured at 120 h in permeabilized cells before and after the addition of 40 μm palmitoyl-l-carnitine chloride supplemented with 0.2 mm malate. The values normalized to cell number are the mean ± S.E. (*n* = 16 per group).

The proximity of methylated lysines 199 and 202 to the recognition loop of ETFβ suggested the possibility that their methylation might influence the interaction of ETFβ with the acyl-CoA dehydrogenases, and thereby affect fatty acid oxidation. Mouse C2C12 myoblasts were employed, as, in contrast to human 143B and HEK cells, they can generate energy from fatty acids. The sequence of DN-(188–205) from mouse ETFβ is identical to that of the bovine and human peptide. In control C2C12 cells, lysine residues 199 and 202 of mouse ETFβ were trimethylated extensively (Fig. 5*A*) and to a greater extent than the methylation of ETFβ in human HEK and 143B cells (Fig. 4, *C* and *E*). Transient suppression of the expression of METTL20 reduced the methylation of ETFβ (Fig. 5*B*) with no effect on the expression of ETFβ. After 48 h, trimethylation had decreased with a concomitant increase in dimethylation, and after 120 h the trimethylation had diminished even further. Mass spectrometric analysis of DN-(188–205) showed that increased suppression of expression of METTL20 from 48 to 120 h was accompanied by decreased methylation of lysines 199 and 202, in comparison with controls (Fig. 5*C*). Thus, the suppression of expression of mouse METTL20 had a similar effect on the methylation of ETFβ to the suppression of the human orthologue.

The role of the methylation of ETFβ on the oxidation of fatty acids in C2C12 cells was examined by measuring the rate of consumption of oxygen in permeabilized mouse C2C12 cells in assay media containing palmitoylcarnitine. At 120 h, suppression of expression of METTL20 was accompanied by a reduction of 14% in the OCR relative to control cells (*t* test, *p* < 0.05), whereas the suppression of expression of ETFβ led to a decrease of 60% in the rate of consumption of oxygen (*t* test, *p* < 0.001) (Fig. 5*D*). No difference in OCR was observed with glutamate-malate, rotenone, succinate, and antimycin A (data not shown). These findings indicate that the trimethylation of lysine residues 199 and 202 of ETFβ by METTL20 influences the oxidation of fatty acids.

## DISCUSSION

### 

#### 

##### Subcellular Site of Methylation of Mitochondrial Proteins

It is conceivable that methylation of the nuclear-encoded proteins found in the matrix of mitochondria or in the inner membranes might take place either during or after cytoplasmic protein synthesis but before import of the protein into the organelle, or during or on completion of import of the protein and cleavage of any mitochondrial targeting sequence. The experiments described here show that METTL20 is a protein lysine methylase associated with human mitochondria, most likely localized to the matrix space, and that it catalyzes the methylation of lysines 199 and 202 in the mitochondrial matrix protein, ETFβ, probably during or following its import into the matrix. Recently, the arginine methyltransferase, NDUFAF7, has also been localized to human mitochondria and shown to methylate Arg-85 of the NDUFS2 subunit in the membrane extrinsic arm of complex I (25). Together, these studies provide the first evidence that both lysine and arginine protein methyltransferases are found in mitochondria, probably in the matrix. With the exception of apocytochrome *c*, which is methylated in the cytoplasm of yeast cells, and the mitochondrial translation release factor MTRF1L methylated by HEMK1 (24), the modifying enzymes, and the subcellular sites of methylation of other mitochondrial proteins remain to be established.

##### Structure and Function of METTL20

**FIGURE 6.**
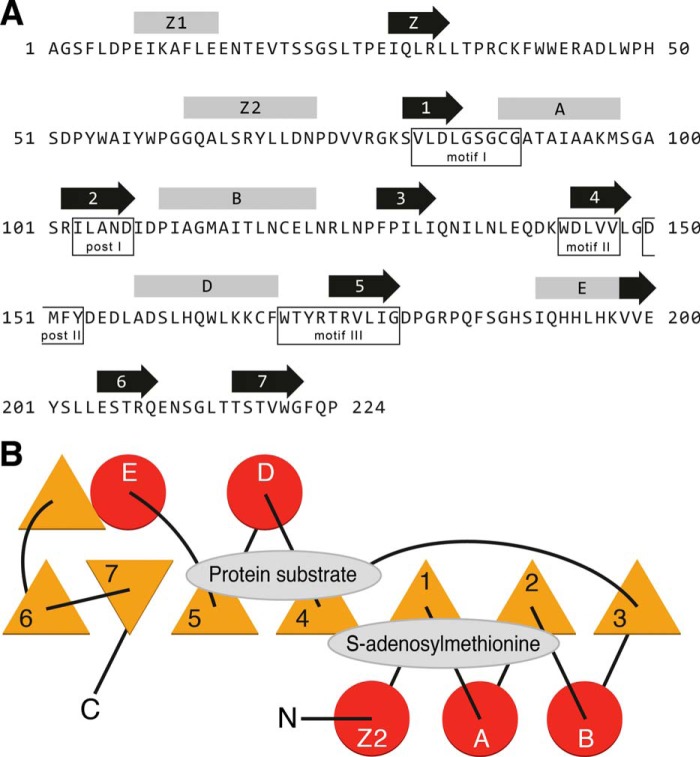
**The structure of human METTL20.** *A*, predicted secondary structures within the sequence of mature METTL20. The locations of α-helices and β-sheets are indicated *above* the sequence by *rods* and *arrows*, respectively. β-Sheets 1–7 and intervening α-helices A-E characterize the 7β-strand methyltransferase-fold. Helix C, intervening between strands 3 and 4 in other family members, is not present in METTL20. Helix Z1 and sheet Z are not part of the fold. Conserved motifs I, post I, II, and III are also found in 7β-strand methyltransferases (56). Motif III consists of β-strand 5 and the amino acid residues preceding it. Post II motif ((D/E)*XX*(Y/F)) is conserved in 9 other related methyltransferases (58). *B*, topology of the 7β-strand fold and flanking α-helices. *Triangles*, *circles*, and *lines* represent β-strands, α-helices, and joining loops, respectively. The positions of the substrate binding sites are shown.

The classification of METTL20 as a 7β-strand methyltransferase (13) provides clues about both its structure and function. This protein family is characterized by a common core similar to a Rossman-fold with a twisted seven-stranded β-sheet structure (strands 1–7 in Fig. 6) with, in the case of METTL20, five associated α-helices (Z2, A, B, D, and E in Fig. 6) rather than the more usual six associated α-helices in other family members. Based on known structures, the site for binding *S*-adenosylmethionine is mainly in loop regions extending from the C-terminal ends of β-sheets 1 and 2, plus conserved sequence motifs I and post I. The region of METTL20 that is likely to be involved in binding the protein substrate, ETFβ, probably involves the regions following β-sheets 4 and 5. Characteristic sequence motifs II and III include these regions (55, 56).

METTL20 has also been grouped in the protein domain family Methyltransf_16 (57, 58) with nine related human proteins that have been proposed to methylate chaperone proteins preferentially. They include METTL18, the possible human homolog of yeast YIL110W, which monomethylates His-243 in ribosomal protein Rpl3 (59), and CaM-KMT, METTL21A, METTL21D, and METTL22, which trimethylate lysines in calmodulin, in HSP70 family members, in valosin-containing protein, and in DNA/RNA-binding protein KIN17, respectively (12, 57, 58, 60). In the present work, analysis of SILAC samples provided clear evidence of association of METTL20 with the ETF. There was much weaker evidence of the possible association of METTL20 with mitochondrial HSP60, which has been reported to contain monomethylated Lys-490, although the extent of methylation was not determined (22). In contrast, in the current experiments, which demonstrate unequivocally the methylation of ETFβ by METTL20, there was no evidence of methylation of HSP60 either under normal growth conditions or when METTL20 was suppressed or overexpressed (data not shown). Therefore, it is unlikely that METTL20 methylates human mitochondrial HSP60.

##### Methylation of the ETF as a Regulatory Mechanism

**FIGURE 7.**
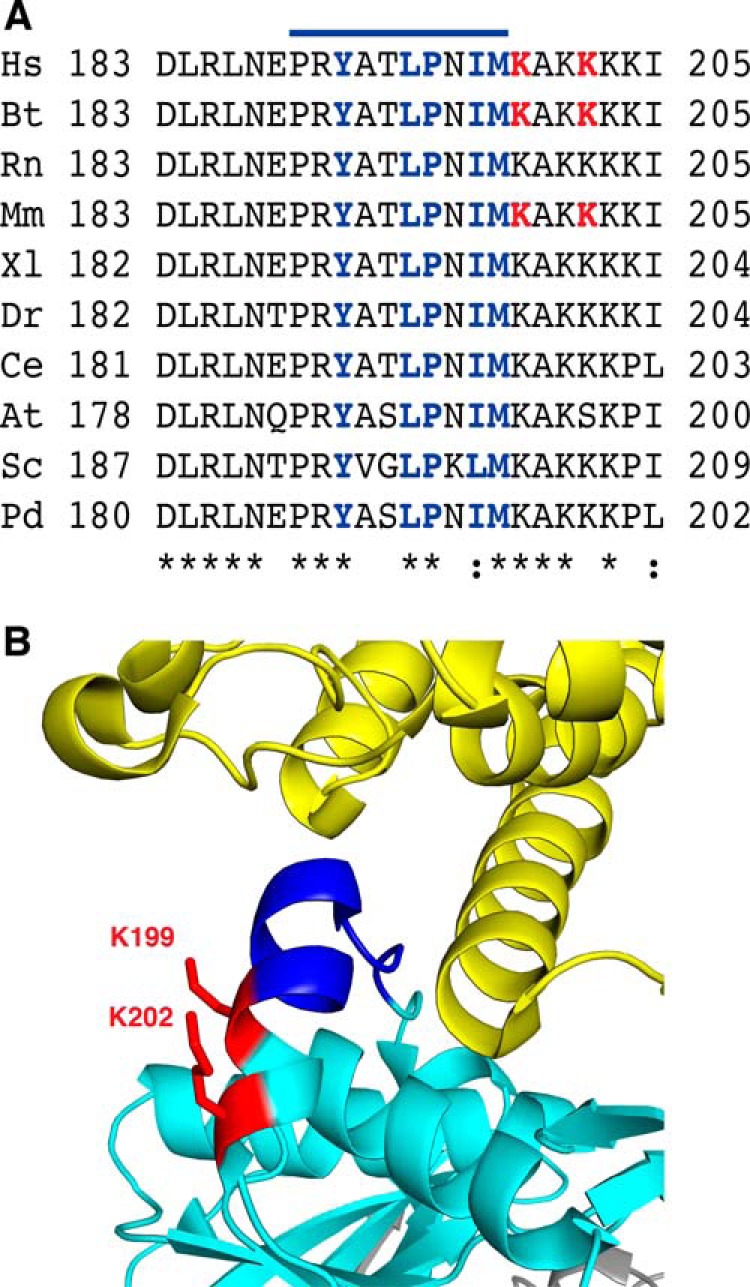
**Proximity of the methylated lysines to the recognition loop in ETFβ.** *A*, conservation of sequences of recognition loops and adjacent residues from representative species. *Hs*, *Homo sapiens*; *Bt*, *Bos taurus*; *Rn*, *Rattus norvegicus*; *Mm*, *Mus musculus*; *Xl*, *Xenopus laevis*; *Dr*, *Danio rerio*; *Ce*, *Caenorhabditis elegans*; *At*, *Arabidopsis thaliana*; *Sc*, *S. cerevisiae; Pd*, *Paracoccus denitrificans*. The symbols (* and :) denote identical and conserved residues, respectively. The *line above* the sequence indicates the position of the recognition loop (33). Residues that interact with dehydrogenases are *blue*, and trimethylated lysines in the human, bovine, and mouse proteins are *red. B*, structure of the region of interaction between recombinant human ETFβ (*turquoise blue*) and the N-terminal region (extended region from residues 10–16 followed by an α-helix from residues 17–46) of the human medium-chain acyl-CoA dehydrogenase (*yellow*). The recognition loop (dark blue) consists of a loop (Pro^189^-Thr^193^) and part of an α-helix (Leu^194^-Met^198^) and is followed immediately by methylated lysines 199 and 202 (*red*).

The trimethylation of a lysine residue changes its properties by conferring a permanent positive charge on its side chain, and by increasing its bulk and hydrophobicity. Thus, the methylation and demethylation of proteins provides a means of modulating interactions between proteins, as exemplified by interactions between histones and their binding partners (3–7). Increasingly, it is becoming recognized as a mechanism for influencing interactions between non-histone proteins also (8, 9, 61), but as yet, there is no evidence for the presence of protein demethylases in mitochondria that might be involved in such a regulatory mechanism. However, the location of methylated lysines 199 and 202 immediately adjacent to the highly conserved recognition loop in ETFβ (Fig. 7*A*) suggested that they may be involved in modulating the interaction of the ETF with dehydrogenases, and possibly with the ETF-ubiquinone oxidoreductase also. The recognition loop has the remarkable property of recognizing and interacting with as many as 13 different acyl-CoA dehydrogenases so as to allow electron transfer between the dehydrogenases and the ETF (30, 31, 34). The structure has been determined of the ETF in complex with one such dehydrogenase, the medium chain acyl-coenzyme A dehydrogenase (33). The recognition loop consists of an extended region from residues 189–193 and the N-terminal region (residues 194–198) of an α-helix, and is followed in the same α-helix (residues 199–203) by methylated lysine residues 199 and 202 (Fig. 7*B*). This structure was determined with unmethylated recombinant human ETF. In the free ETF, Lys-199 and Lys-202 would be in exposed positions, where they would be amenable to methylation by METTL20, whereas lysines 201, 203, and 204 are buried and are not methylated. The introduction of up to six collectively bulky methyl groups in lysines 199 and 202 would be likely to alter the interaction of the ETF with the dehydrogenase. ETFβ interacts also with the ETF-ubiquinone oxidoreductase bound in the inner mitochondrial membrane (36). Hitherto, no structure has been described of the complex between the ETF and the ubiquinone oxidoreductase, and so it is not known whether their interaction also involves the recognition loop and lysine residues 199 and 202.

In contrast to 143B and HEK cells, C2C12 cells are able to respire using fatty acids. In the present work, the extent of methylation of ETFβ was demonstrably higher in C2C12 cells compared with 143B and HEK cells, which implies it has a relevant biological function depending on the energetic substrates. Furthermore, the reduction of methylation of lysine residues 199 and 202 by suppression of METTL20 in C2C12 cells that are oxidizing palmitoylcarnitine decreased its oxidation. Therefore, the methylation of these lysines influences fatty acid oxidation probably by modulating protein-protein interactions involving ETFβ. Such a mechanism could be involved in the formation of a multifunctional fatty acid oxidation complex that was reported to interact with OXPHOS supercomplexes (62).

Mutations in ETFα and ETFβ and in the ETF-ubiquinone oxidoreductase are associated with the human inherited metabolic disease, glutaric acidemia type II or multiple acyl-CoA dehydrogenase deficiency (63, 64), and deletion of Lys-201 in mature ETFβ has been observed in a patient suffering from ETF deficiency (65). It was postulated that the mutation disrupts the local structure and impacts on the ETF partner complex formation due to the close proximity of Lys-201 to the recognition loop (33). It remains to be established whether mutations in METTL20 that would affect the methylation of lysine residues 199 and 202 of ETFβ are also associated with metabolic diseases.

## Footnotes

3 The abbreviations used are:

HEMK1: heme biosynthesis gene K
ETD: electron transfer dissociation
ETF: electron transfer flavoprotein
METTL12: methyltransferase-like protein 12
METTL20: methyltransferase-like protein 20
NDUFAF7: NADH dehydrogenase (ubiquinone) complex I assembly factor 7
NDUFB3: NADH dehydrogenase (ubiquinone) 1β subcomplex subunit 3
NDUFS2: NADH dehydrogenase (ubiquinone) iron-sulfur protein 2
OCR: oxygen consumption rate
SILAC: stable isotope labeling by amino acids in cell culture
TML: trimethyllysine
DML: dimethyllysine.
